# Integrated Random Negative Sampling and Uncertainty Sampling in Active Learning Improve Clinical Drug Safety Drug–Drug Interaction Information Retrieval

**DOI:** 10.3389/fphar.2020.582470

**Published:** 2021-04-23

**Authors:** Weixin Xie, Limei Wang, Qi Cheng, Xueying Wang, Ying Wang, Hongyuan Bi, Bo He, Weixing Feng

**Affiliations:** ^1^ Institute of Intelligent System and Bioinformatics, College of Intelligent Systems Science and Engineering, Harbin Engineering University, Harbin, China; ^2^ Key Laboratory of Tropical Translational Medicine of Ministry of Education, College of Biomedical Information and Engineering, Hainan Medical University, Haikou, China; ^3^ The Fourth Affiliated Hospital of Harbin Medical University, Harbin, China

**Keywords:** drug–drug interaction (DDI), information retrieval (IR), text mining (TM), active learning (AL), random negative sampling

## Abstract

Clinical drug–drug interactions (DDIs) have been a major cause for not only medical error but also adverse drug events (ADEs). The published literature on DDI clinical toxicity continues to grow significantly, and high-performance DDI information retrieval (IR) text mining methods are in high demand. The effectiveness of IR and its machine learning (ML) algorithm depends on the availability of a large amount of training and validation data that have been manually reviewed and annotated. In this study, we investigated how active learning (AL) might improve ML performance in clinical safety DDI IR analysis. We recognized that a direct application of AL would not address several primary challenges in DDI IR from the literature. For instance, the vast majority of abstracts in PubMed will be negative, existing positive and negative labeled samples do not represent the general sample distributions, and potentially biased samples may arise during uncertainty sampling in an AL algorithm. Therefore, we developed several novel sampling and ML schemes to improve AL performance in DDI IR analysis. In particular, random negative sampling was added as a part of AL since it has no expanse in the manual data label. We also used two ML algorithms in an AL process to differentiate random negative samples from manually labeled negative samples, and updated both the training and validation samples during the AL process to avoid or reduce biased sampling. Two supervised ML algorithms, support vector machine (SVM) and logistic regression (LR), were used to investigate the consistency of our proposed AL algorithm. Because the ultimate goal of clinical safety DDI IR is to retrieve all DDI toxicity–relevant abstracts, a recall rate of 0.99 was set in developing the AL methods. When we used our newly proposed AL method with SVM, the precision in differentiating the positive samples from manually labeled negative samples improved from 0.45 in the first round to 0.83 in the second round, and the precision in differentiating the positive samples from random negative samples improved from 0.70 to 0.82 in the first and second rounds, respectively. When our proposed AL method was used with LR, the improvements in precision followed a similar trend. However, the other AL algorithms tested did not show improved precision largely because of biased samples caused by the uncertainty sampling or differences between training and validation data sets.

## 1 Introduction

The concurrent use of multiple drugs (i.e., polypharmacy) has become increasingly common around the world, and its prevalence also has increased. This is attributable to multiple comorbidities and/or preventative care, which necessitate multiple drug treatments (Ward et al., 2014; Kantor et al., 2015; Qato et al., 2016). Currently, a significant number of people use one or multiple preventative medications; for instance, most cancer survivors are prescribed five or more drugs, including drugs to prevent cancer first occurrence or recurrence (Murphy et al., 2018). However, polypharmacy has significantly increased the likelihood of drug–drug interaction (DDI) combinations (Percha and Altman, 2013; Patel et al., 2014), which can lead to many negative clinical consequences, including ADEs, especially in seniors (Budnitz et al., 2006; Bourgeois et al., 2010). Literature review and meta-analysis showed that drug interactions caused 1.1% hospital admissions and 0.54% of the emergency department visits in the United States (Becker et al., 2007; Magro et al., 2012; Dechanont et al., 2014), which means that DDIs increase the cost to society and can become a great threat to the public health system (Classen et al., 1997; Eltyeb and Salim, 2014). More seriously, some DDIs could potentially be very dangerous and even cause death (Bates et al., 1995). Therefore, identifying clinically significant DDIs has high potential in preventing ADEs, and in reducing morbidity and mortality as well as healthcare costs. A large amount of DDI evidence is hidden in the text of biomedical literature. Therefore, with increasing rates of polypharmacy, DDI information retrieval from the literature is a vital part of preventing clinically significant DDIs and ADEs.

Text mining has been used widely to identify and extract DDI data from biomedical literature (Thamrongrattanarit et al., 2012; Percha et al., 2012). Several DDI corpora have been developed. The DDI extraction challenge tasks (DDI-ECT) in 2011 and 2013 (Bedmar and Martinez, 2011; Segura-Bedmar et al., 2014) provided the pharmacological substances and DDI relationships in sentences in DrugBank and PubMed abstracts (Herrero-Zazo et al., 2013). The pharmacokinetic DDI package insert corpus (PK-DDI-PI) (Boyce et al., 2012) provided a second body of information regarding evidence of drug interactions. It was built using FDA-approved drug labels, and focused exclusively on annotating DDI relationships. The pharmacokinetic corpus developed by our group contained four classes of pharmacokinetic abstracts, including *in vivo* pharmacokinetics, *in vivo* pharmacogenetics, *in vivo* DDI, and *in vitro* DDI studies (Wu et al., 2013). Many text mining methods based on machine learning algorithms have been applied to detect DDI-relevant sentences (i.e., detection) and classify interacting drugs (i.e., classification: classifying into one of four types “advise,” “effect,” “mechanism,” and “int”) on the corpora, especially on DDI-ECT (Ananiadou et al., 2006; Segura-Bedmar et al., 2014). Kim et al. (2015) used a support vector machine (SVM) model and achieved competitive performances for DDI detection (F1 = 0.775) and DDI classification (F1 = 0.670). Zheng et al. (2016) applied a context vector graph kernel to the DDI-ECT 2013 corpus and achieved acceptable performances for DDI detection (F1 = 0.818) and DDI classification (F1 = 0.684). Various deep neural network algorithms also have been proposed for DDI classification on the DDI-ECT 2013 corpus (Liu et al., 2016). Zhao et al. (2016) proposed a syntax convolutional neural network that combined a traditional convolutional neural network and external features, such as contexts, shortest path, and part-of-speech, to extract and classify DDIs. It obtained F1-scores of 0.772 and 0.686 for DDI detection and classification, respectively. By integrating a recurrent neural network with multichannel word embedding, Zheng et al. (2017) combined an attention mechanism and a recurrent neural network with long short-term memory units and obtained a system that performed well for DDI detection (F1 = 0.840) and classification (F1 = 0.773). Zhang et al. (2018) proposed a hierarchical recurrent neural network-based method to integrate the shortest dependency paths and sentence sequence, which produced an F1-score of 0.729 for DDI classification. Wang et al. (2017) introduced the dependency-based technique to a bidirectional long short-term memory network and built linear, DFS (depth-first search), and BFS (breadth-first search) channels, which made significant progress in DDI detection and gave an F1-score of 0.720 for DDI classification. Deng et al. (2020) proposed a multimodal deep learning framework named DDIMDL to predict DDI-associated events, and the accuracy of the model achieved 0.8852.

IR systems aim to retrieve the most relevant articles to the given queries and meanwhile address the diversity of search results for completely meeting the information needs (Hersh et al., 2006; Hersh and Voorhees, et al., 2009). In this article, we focus on IR methods for detecting clinical drug safety DDI-relevant abstracts from PubMed. The goal of IR applying is transformed to retrieve clinical drug safety DDI-relevant documents covering as many aspects of the query as possible. An aspect of a query can be described with certain biomedical terms (Xu et al., 2019). This task differs from previously described text mining methods that focused on detecting DDI-relevant sentences or classifying drug interaction pairs. Two DDI IR studies have been reported previously. Kolchinsky et al. (2015) implemented a set of classifiers (variable trigonometric threshold (VTT), SVM, logistic regression, Naïve Bayes, linear discriminant analysis (LDA), and dLDA) to identify abstracts relevant to pharmacokinetic DDI evidence. Their work contributed to the construction of a corpus of pharmacokinetic-related abstracts that is publicly available as “Pharmacokinetic DDI-Relevant Abstracts V0” (Wu et al., 2013). They discovered that bigram features were important for classification. Among the classifiers, LDA achieved the best performance (F1 = 0.93). Zheng et al. (2019) presented a DDI-PULearn (positive-unlabeled learning) method for large-scale DDI predictions; it obtained F1-scores of 0.860 and 0.862 for 3-fold and 5-fold cross-validation, respectively. Using a different abstract corpus that contained DDI-relevant, drug-relevant but DDI-irrelevant, and drug-irrelevant abstracts from PubMed, Zhang et al. (2017) developed a two-stage cascade classifier to separate DDI-relevant abstracts from abstracts that were either drug-irrelevant or drug-relevant but DDI-irrelevant. Their classifier addressed the issue of class imbalance in DDI retrieval, and its performance measures were precision = 0.83, recall = 0.95, and F1 = 0.88.

Although machine learning (ML) approaches can effectively detect DDI-relevant abstracts, they usually require a large number of high-quality samples manually curated by domain experts, which is highly time consuming. Active learning (AL) is an interactive approach that guides new training sample selection and updates ML algorithm training that previous ML algorithms performed poorly. AL significantly reduced the total training sample size and therefore led to less manual data curating (Lewis and Catlett, 1994; Settles and Craven, et al., 2008; Lewis and Gale et al., 1994). Several AL applications in biomedical research are available. For instance, Bressan et al. (2019) proposed the selection criteria based on representativeness and uncertainty sampling to explore the most informative samples in six public biomedical datasets, and obtained 97% accuracy with just 24 samples. Another AL application is the phenotyping algorithm that was developed using electronic health records. Chen et al. (2013) investigated how AL performed in developing phenotyping algorithms for two phenotypes (rheumatoid arthritis and colorectal cancer) and demonstrated that, compared with the ML approach, AL reduced the number of annotated samples required to achieve an AUC of 0.95 by 68% (rheumatoid arthritis) and 23% (colorectal cancer) when unrefined features were used in the two phenotypes.

The essential idea of AL is uncertainty sampling. In each round, the ML algorithm is trained and updated based on the samples with the highest uncertainty (i.e., uncertainty sampling) predicted from the current ML algorithm (Lewis and Catlett et al., 1994). Because AL directs ML to concentrate its training on the low-performance samples through uncertainty sampling, it effectively reduces the sample size, and hence the manual curating cost (Chen et al., 2013). DDI-related abstracts are only a very small fraction of the total 30 million abstracts in PubMed, especially the abstracts related to clinical drug safety DDIs. Although some DDI corpora have already been developed in recently years, the consistency of their content is limited (Herrero-Zazo et al., 2013). The DDI corpus regarding clinical drug safety differs from existing corpora in the scope of the annotated DDIs, while it is annotated with a specific DDI category. Therefore, a random sample subset of PubMed abstracts will be largely negative, that is, non–DDI-related. This random negative sampling scheme is a very cheap way of collecting negative samples that do not need manual curating. The present study aimed to investigate how uncertainty sampling and random negative sampling can be integrated into AL to achieve an optimal DDI IR performance. In the AL research literature, the selection of external validation samples has not been well studied. We considered that the new positive and negative samples that AL guided and collected would be different from the positive and negative samples that were collected initially. Therefore, when we develop an AL algorithm and demonstrate its performance improvement, we will update not only the training data but also the external validation data set accordingly.

## 2 Materials and Methods

### 2.1 Data Source

We constructed a clinical drug safety DDI corpus in this study. First, PubMed abstracts were screened with a keyword query: [“drug interaction” AND (Type of Study)] and [“drug combination” AND (Type of Study)]. Then, 600 positively labeled and 400 negatively labeled clinical drug safety DDI abstracts were selected using the inclusion and exclusion criteria detailed in Table 1.

**TABLE 1:** Inclusion and exclusion criteria for clinical drug safety drug–drug interaction (DDI) abstract selection.

Inclusion	Clinical trial DDI toxicity study: Phase I/II/III clinical trials in which drug combination and/or single drug toxicity data are evaluated and reported
	Pharmaco-epidemiological DDI study: Pharmaco-epidemiology studies in which toxicities from drug combinations are reported and compared to toxicities from a single drug
	DDI and adverse drug event (ADE) case reports: DDI-induced ADE cases in which the time sequential drug and ADE are reported in clinical settings
Exclusion	a) Clinical PK DDI study: Both single drug and drug combination exposures (i.e., pharmacokinetics) are evaluated either in patients or healthy volunteers
	b) Clinical PK PG study: The single drug exposure (i.e., pharmacokinetics) is evaluated among patients who have different genotypes in CYP450 and UGT enzymes and drug transporters
	c) *In vitro* PK study: Substrate depletion and metabolite formation study are for the *fm* data collection, and inhibition study is for the *Ki* data collection
	d) Drug interaction detection algorithms or software
	e) Compliance of avoiding DDIs
	f) Concordance of DDI reporting among different drug interaction knowledge base
	g) Comparison of tde performance of DDI clinical decision systems
	h) Drug–alcohol/food interactions
	i) Drug/test interactions
	j) Case report studies
	k) Review papers
	l) Cell culture and animal studies
	m) Other studies that are not related to drug interactions

In addition, we randomly selected 9,200 abstracts from PubMed. Unlike the 600 positive and 400 negative drug safety DDI abstracts, these abstracts were not manually reviewed or labeled. Assuming most of the 9,200 abstracts were negative, we considered that as random negative samples.

### 2.2 Preprocessing and Feature Selection

**FIGURE 1:**
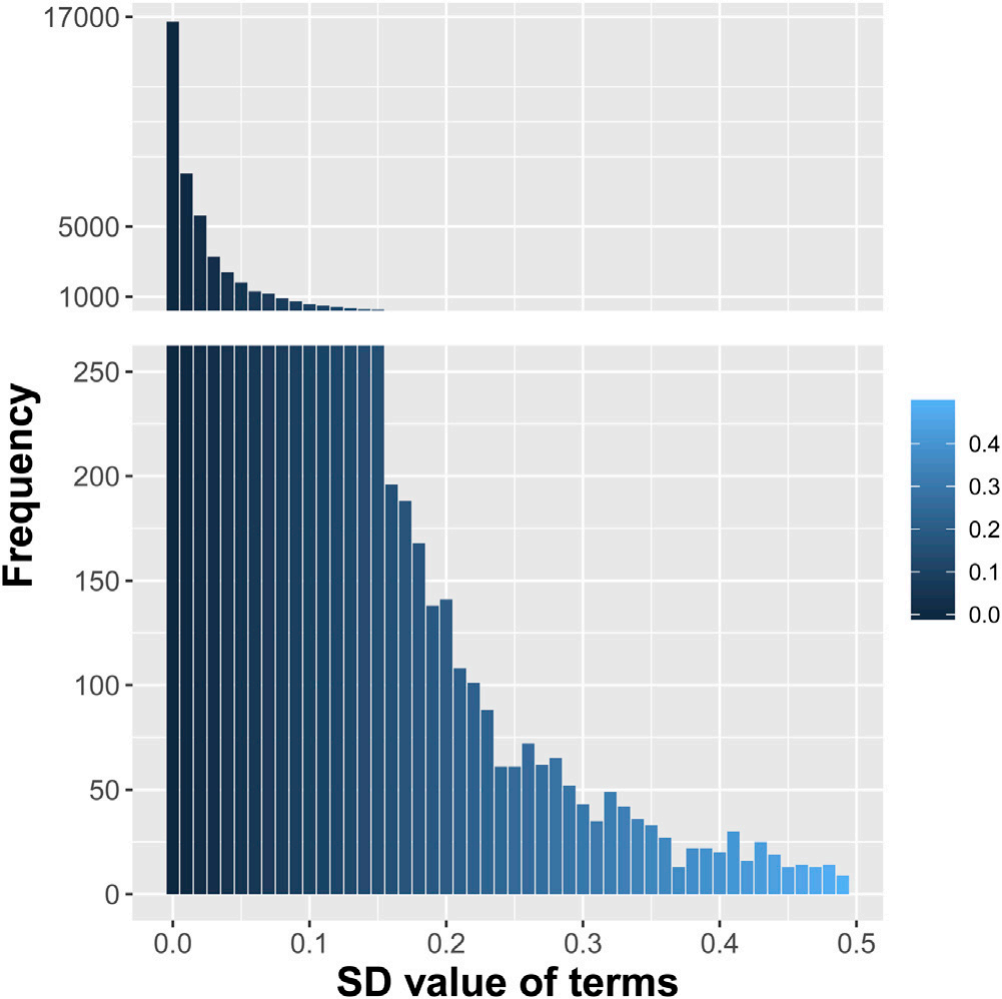
Distribution of the standard deviations (SDs) of the initial 46,604 terms from all the selected abstracts.

**FIGURE 2:**
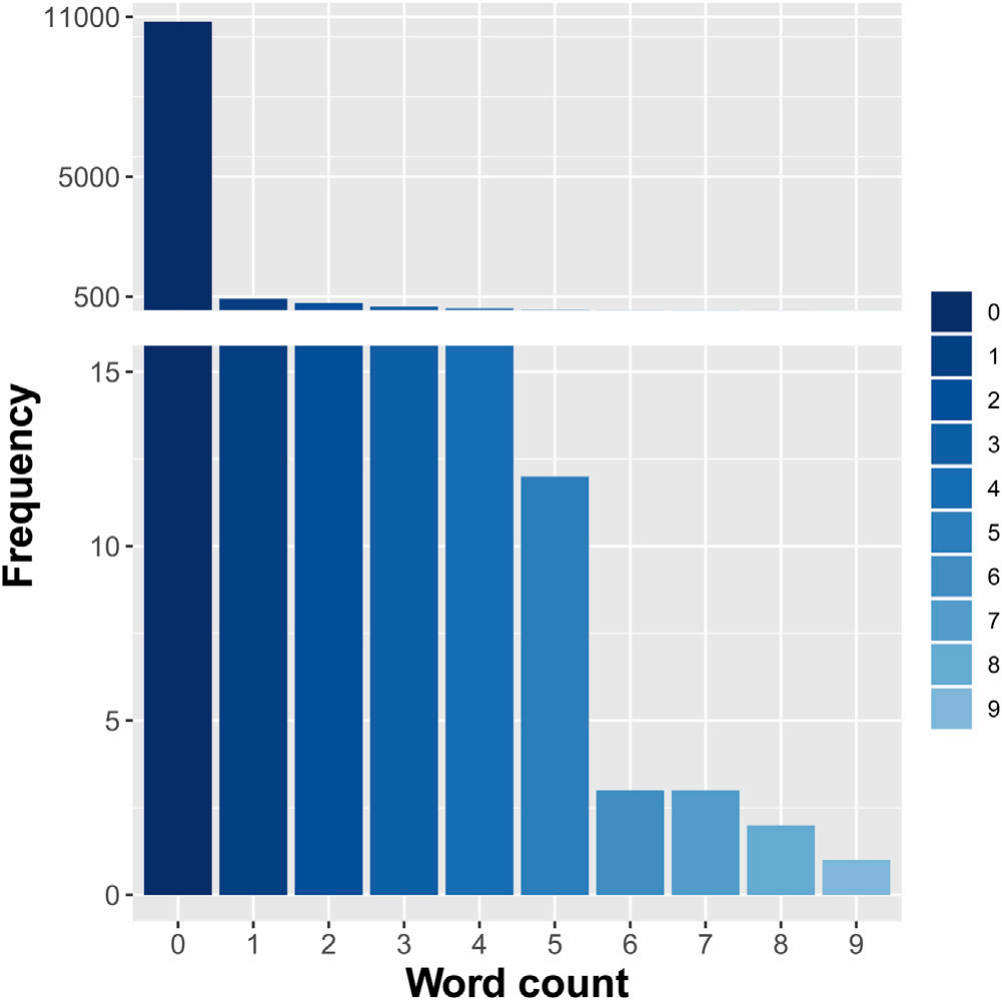
Frequency distribution of one representative term (“advanc”) in all the selected texts.

All the selected abstracts were retrieved from PubMed. After a series of processing including parsing the desired content (titles and abstracts), converting to GENIA format, and converting to multiple text files, the abstracts were saved in text format. And the word stemming was implemented to remove the common morphological and inflectional endings from words, and to map the related words to the same stem. This process erased word suffixes to retrieve their radicals. We used the “tm” text mining package in R for word stemming and obtained each word’s stem with its suffixes removed. Then, initialization and normalization processes were implemented to convert lowercase letters, remove punctuation, and create term–document matrices. A total of 46,604 terms were created from all the abstracts including titles. The frequency distribution of the standard deviations (SDs) of these terms was shown in Figure 1. The frequency of appearance of each term in the samples followed Poisson distribution and was represented as a categorical term–document occurrence matrix based on the word count. The terms with low SDs were considered to lack useful information and specificity. Besides, the frequencies of terms with SDs ≤0.03 were more than 5,000, which meant they were many repeats in a lot of abstracts. Therefore, the 16,200 terms with SDs >0.03 were selected as features. The distribution of one representative term (word stem: “advanc”) in all the texts is shown in Figure 2.

### 2.3 Active Learning Methods for DDI IR

In this study, we investigated four different AL methods. We began with the conventional and simplest AL method, which had a single ML algorithm with uncertainty sampling and no validation sample update. The second AL method added random negative sampling to the uncertainty sampling and had a single ML algorithm and no validation sample update. The third AL method added a second ML algorithm for random negative sampling but had no validation sample update. The fourth AL method was similar to the third method but included a validation sample update. The training and validation sample sizes for the four AL algorithms are shown in Table 2.

**TABLE 2:** Training and validation sample sizes for four different active learning (AL) methods in two rounds.

AL algorithms		First round (SVM)		Second round (SVM)		First round (LR)		Second round (LR)	
		Training	Validation	Training	Validation	Training	Validation	Training	Validation
Traditional AL		200+* 200-*	200+* 200-*	208+* 567-*	200+* 200-*	200+* 200-*	200+* 200-*	205+* 473-*	200+* 200-*
Traditional AL with random negative sampling		200+* 1000R*	200+* 200-* 200R*	217+* 1104R*	200+* 200-*200R*	200+* 1000R*	200+* 200-* 200R*	211+* 1085R*	200+* 200-* 200R*
AL with two separate ML algorithm integrated random negative sampling	ML_1_	200+* 1000R*	200+* 200-* 200R*	209+* 1213R*	200+* 200-* 200R*	200+* 1000R*	200+* 200-* 200R*	206+* 1106R*	200+* 200-* 200R*
	ML_2_	200+* 200-*		209+* 412-*		200+* 200-*		206+* 306-*	
AL with two separate ML algorithm integrated random negative sampling, and validation sample update	ML_1_	200+* 1000R*	209+* 412-* 196R*	204+* 1053R*	209+* 412-* 196R*	200+* 1000R*	204+* 391-* 199R*	202+* 1046R*	204+* 391-* 199R*
	ML_2_	200+* 200-*		205+* 306-*		200+* 200-*		202+* 295-*	

Note: +* (positive samples), -*(negative samples), and R*(random negative samples).

#### 2.3.1 Traditional AL

**FIGURE 3:**
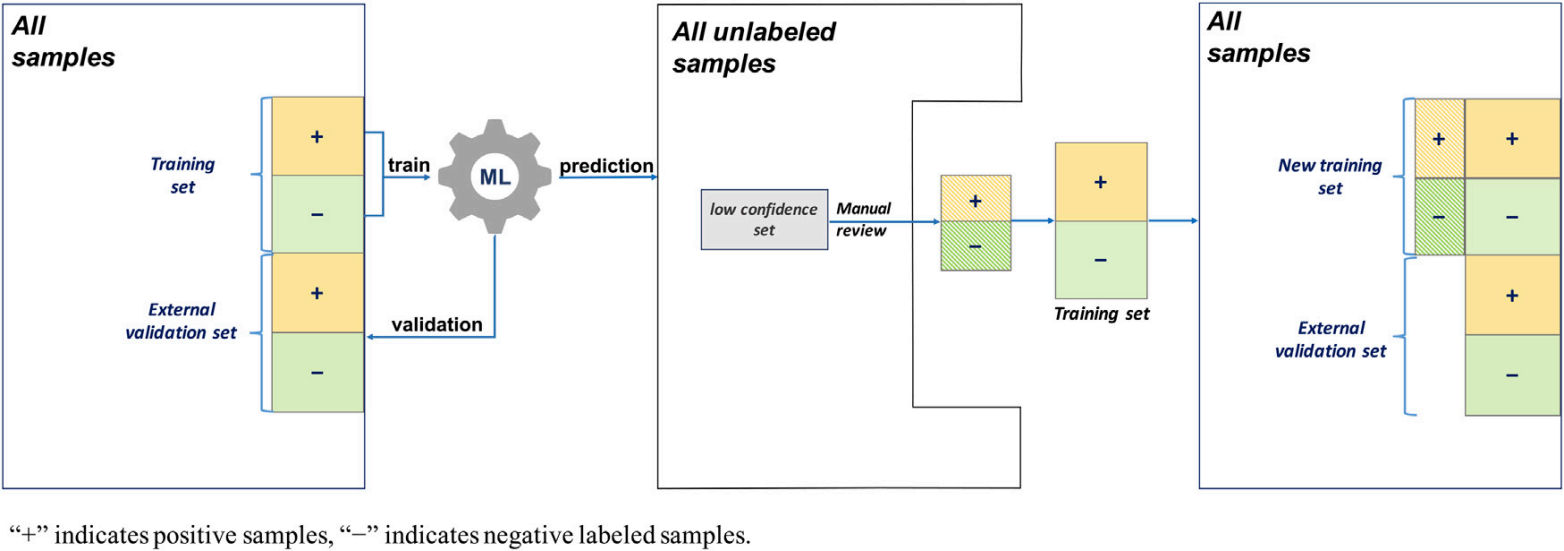
Active learning workflow with a single machine learning (ML) algorithm, uncertainty sampling, and no validation update.

The AL workflow and related training and validation data sets are presented in Figure 3. During the first round, a single ML algorithm was trained using 200 positively labeled and 200 negatively labeled abstracts (see Table 2). Its performance was evaluated using 200 positive and 200 negative abstracts from the external validation data set. Then, to predict the unlabeled 9,200 abstracts that were randomly selected from PubMed, a random subset of low confidence samples (i.e., uncertainty sampling with classification probability between 0.4 and 0.6) was extracted. They were reviewed manually, labeled, and added back to the training set for the second round ML algorithm training. The same external validation data were used in the second evaluation round.

#### 2.3.2 Traditional AL With Random Negative Sampling

**FIGURE 4:**
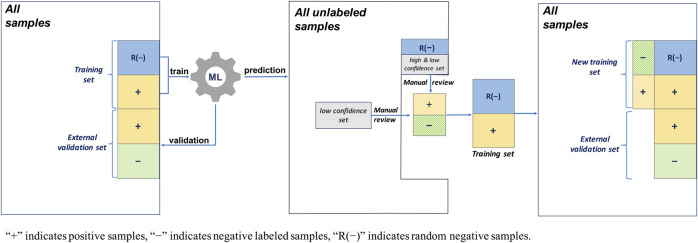
Active learning workflow with a single machine learning (ML) algorithm, uncertainty sampling plus random negative sampling, and no validation update.

This AL workflow and related training and validation data sets are presented in Figure 4. Unlike the first AL method, this AL method had 1,000 random negative samples as the negative abstracts (see Table 2). In the first round, the ML algorithm was trained using 200 positively labeled abstracts and 1,000 random negative samples. Its performance was evaluated using 200 positive and 200 negative abstracts, and 200 random negative samples from the external validation set. Then, to predict the 9,200 unlabeled samples, including the random negative samples in the first round, a random subset of low confidence samples (i.e., uncertainty sampling with a probability between 0.4 and 0.6) was extracted. We also included high confidence positively predicted abstracts (probability >0.7) in the random negative samples. These two parts of uncertainty samples were reviewed manually and labeled, and then they were added back to the initial training set. The new training set was for the second round ML algorithm training. The external validation data were the same in the two rounds of evaluation.

#### 2.3.3 AL With Two Separate ML Algorithms Integrated Random Negative Sampling

**FIGURE 5:**
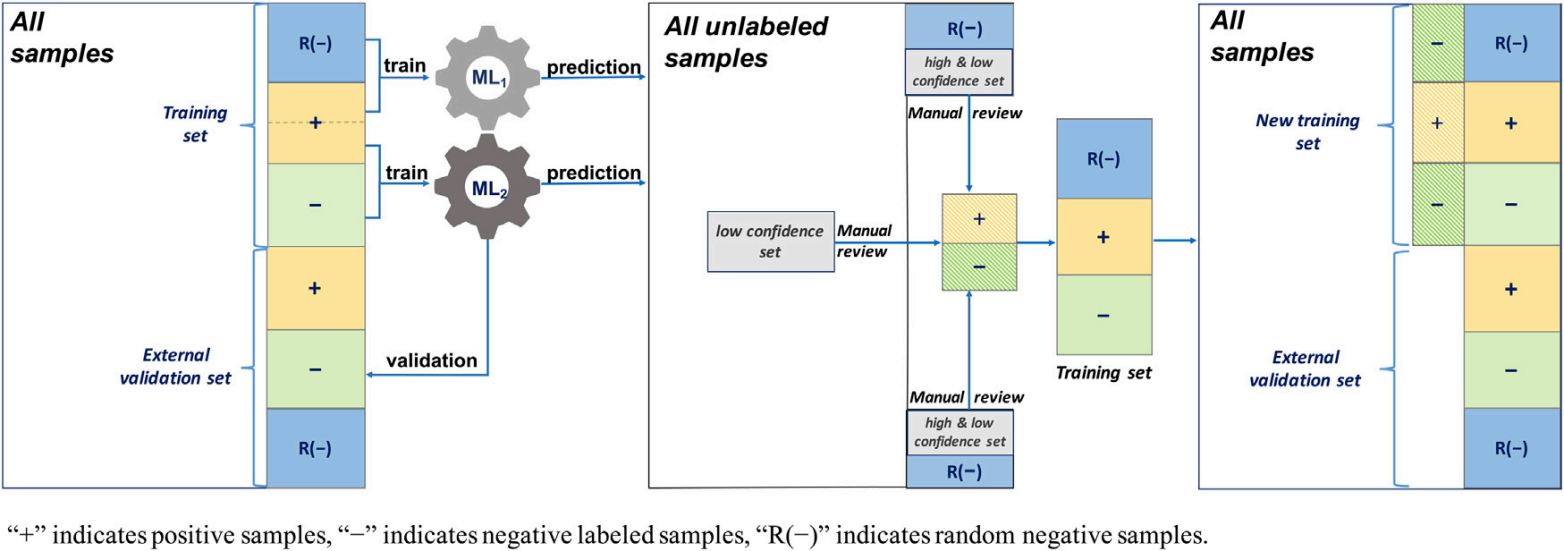
Active learning workflow with two separate ML algorithms, uncertainty sampling plus random negative sampling, and no validation update.

This AL workflow and related training and validation data sets are shown in Figure 5. This AL also had 1,000 negative random samples as part of the negative abstracts, but, unlike the second AL, two separate ML algorithms were constructed. In the first round, ML_1_ was trained using 1,000 random negative samples and 200 positively labeled abstracts, whereas ML_2_ was trained using 200 positively labeled and 200 negatively labeled abstracts (see Table 2). Their performances were evaluated using 200 positively labeled and 200 negatively labeled abstracts, and 200 random negative samples. Then, to predict all the unlabeled samples, including the random negative samples in the first round, a random subset of the low confidence samples (i.e., uncertainty sampling with a probability between 0.4 and 0.6) was extracted. We also included high confidence positively labeled abstracts (probability >0.7) in the random negative samples. These abstracts were labeled by manual review and added back to two initial training sets. The same external validation data were used in the second evaluation round.

#### 2.3.4 AL With Two Separate ML Algorithms Integrated Random Negative Sampling and Validation Sample Update

**FIGURE 6:**
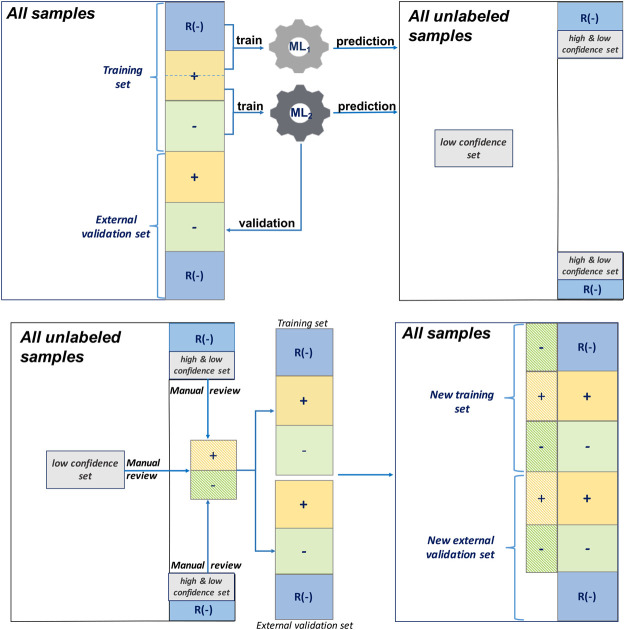
Active learning workflow with two separate ML algorithms, uncertainty sampling plus random negative sampling, and validation sample update.

This AL method is the one proposed in this study. The proposed AL workflow and related training and validation data sets are presented in Figure 6. The following is a detailed description.

- Two training sets and an external validation set: One training set contained 1,000 random negative samples and 200 positively labeled abstracts, and the other contained 200 positively labeled and 200 negatively labeled abstracts. The initial validation set was the same as that used for the third AL, namely, 200 positively labeled and 200 negatively labeled abstracts, and 200 random negative samples (see Table 2).
- First-round ML training: ML_1_ and ML_2_ were built separately with the training sets.
- Uncertainty sampling: To predict all 9,200 unlabeled samples, including the random negative samples in the first round, a random subset of the low confidence samples (probability between 0.4 and 0.6) was extracted. We also included high confidence positively predicted abstracts (probability >0.7) in the random negative samples.
- Manual review and re-splitting data into training and validation data sets: All new samples selected from the uncertainty sampling were labeled by manual review and divided into three parts, and then added back into the two initial training sets and external validation set.
- Second round ML training: ML_1_ and ML_2_ were built separately using the updated training sets.
- Model performance evaluation: The performances of the first and second round MLs were evaluated using the updated external validation data sets.

### 2.4 Evaluation

We used SVM and LR as the primary ML algorithms to evaluate the four AL methods. Table 2 shows the training sets and validation sets of SVM and LG in the two rounds of AL processing. The performances of four AL methods were evaluated using recall (R) = TP/(TP + FN), precision (P) = TP/(TP + FP), and F1-score by confusion matrix. The F1-score was defined as F1-score = (2×P×R)/(P + R). We reported P when R was set as 0.99 that was utilized to present the precision of models when most positive samples were identified. We expected to reduce the occurrence of the false positives when detecting DDI toxicity information in the literature, and preferred the improved precision value while maintaining the highest R.

## 3 Results

### 3.1 Traditional ALs Gave Decreased Performances in the Second Round

When we used SVM, the performance of the traditional AL method, that is, uncertainty sampling with a single ML algorithm, reduced from the first to the second round (R, P, F1) = (0.95, 0.99, 0.97) to (0.67, 1.00, 0.80) (Table 3). When R was set as 0.99, P decreased from 0.97 to 0.94. When we used LR, the performance from the first to the second round presented (R, P, F1) = (0.94, 0.97, 0.95) to (0.81, 0.99, 0.89), and P decreased from 0.96 to 0.94 when R = 0.99.

**TABLE 3:** Performances (SVM and LR) of two active learning methods with single machine learning (ML) algorithms.

Two AL methods’ performances for SVM		TP	FP	TN	FN	Recall	Precision	F-score	Precision (recall = 0.99)
Uncertainty sampling, a single ML algorithm, and no validation data update	1st round	190	2	198	10	0.95	0.99	0.97	0.97
	2nd round	134	0	200	66	0.67	1.00	0.80	0.94
Uncertainty sampling and random negative sampling, a single ML algorithm, and no validation data update	1st round	176	0	400	24	0.88	1.00	0.94	0.96
	2nd round	138	0	400	62	0.69	1.00	0.82	0.94
**Two AL methods’ performances for LG**		**TP**	**FP**	**TN**	**FN**	**Recall**	**Precision**	**F-score**	**Precision (Recall = 0.99)**
Uncertainty sampling, a single ML algorithm, and no validation data update	1st round	187	7	193	13	0.94	0.97	0.95	0.96
	2nd round	161	1	199	39	0.81	0.99	0.89	0.94
Uncertainty sampling and random negative sampling, a single ML algorithm, and no validation data update	1st round	176	5	395	24	0.88	0.97	0.92	0.95
	2nd round	159	1	399	41	0.80	0.99	0.88	0.94

The performance of the second AL method for SVM also reduced from the first to second rounds (R, P, F1) = (0.88, 1.00, 0.94) to (0.69, 1.00, 0.82) (Table 3), even when random negative samples were added to the negatively labeled abstracts in either the training or validation data. When R was set as 0.99, P decreased from 0.96 to 0.94. The performance for LR showed the same trends (R, P, F1) = (0.88, 0.97, 0.92) to (0.80, 0.99, 0.88). When R was set as 0.99, P decreased from 0.95 to 0.94.

Considering the negatively labeled abstracts and random negative samples are different, we implemented two separate ML algorithms in the third AL algorithm (Table 4). Appling SVM, the performance of ML_1_, which was designed to differentiate random negative samples from positive samples, reduced from the first to second rounds (R, P, F1) = (0.89, 1.00, 0.94) to (0.66, 1.00, 0.80). The performance of ML_2_, which was constructed to differentiate negatively labeled abstracts from positive samples, also reduced from the first to second rounds (R, P, F1) = (0.95, 0.97, 0.96) to (0.75, 1.00, 0.86). With LR as the algorithm, the performance (R, P, F1) of ML_1_ and ML_2_ reduced from (0.90, 0.98, 0.94) to (0.71, 0.99, 0.83) and (0.91, 1.00, 0.95) to (0.76, 0.99, 0.86), respectively. When setting R as 0.99, none of their P was improved in the second round.

**TABLE 4:** Performance (SVM and LR) of an active learning method with two separate machine learning (ML) algorithms.

Performance			TP	FP	TN	FN	Recall	Precision	F-score	Precision (Recall = 0.99)
Uncertainty sampling and random negative sampling, two separate ML algorithms, and no validation data update (SVM)	ML_1_	1st round	178	0	400	22	0.89	1.00	0.94	0.96
		2nd round	132	0	400	68	0.66	1.00	0.80	0.94
	ML_2_	1st round	190	6	394	10	0.95	0.97	0.96	0.94
		2nd round	150	0	400	50	0.75	1.00	0.86	0.94
Uncertainty sampling and random negative sampling, two separate ML algorithms, and no validation data update (LG)	ML_1_	1st round	180	3	397	20	0.90	0.98	0.94	0.96
		2nd round	142	2	398	58	0.71	0.99	0.83	0.93
	ML_2_	1st round	182	0	400	18	0.91	1.00	0.95	0.95
		2nd round	152	2	398	48	0.76	0.99	0.86	0.94

### 3.2 The Proposed AL Method Performed Well in the Second Round

We considered that the reduced performances of the previous three AL methods in the second round may be because the validation samples were incompatible with the training samples after the AL step. Therefore, in our proposed AL method with two separate ML algorithms, we updated the validation samples. The performance of our AL method was presented in Table 5. ML_1_ was designed to differentiate random negative samples from positive samples. ML_2_ was constructed to differentiate negatively labeled abstracts from positive samples.

**TABLE 5:** Performance of our proposed active learning (AL) method evaluated using SVM and LR.

Performance			TP	FP	TN	FN	Recall	Precision	F-score	Precision (Recall = 0.99)
AL with two separate ML algorithms integrated random negative sampling, and validation sample update (SVM)	ML_1_	1st round	182	6	602	27	0.87	0.97	0.92	0.70
		2nd round	178	0	608	31	0.85	1.00	0.92	0.82
	ML_2_	1st round	199	98	510	10	0.95	0.67	0.79	0.45
		2nd round	184	4	604	25	0.88	0.98	0.93	0.83
AL with two separate ML algorithms integrated random negative sampling, and validation sample update (LG)	ML_1_	1st round	159	8	582	45	0.78	0.95	0.86	0.81
		2nd round	161	18	572	43	0.79	0.90	0.88	0.84
	ML_2_	1st round	194	58	532	10	0.95	0.77	0.85	0.60
		2nd round	188	6	584	16	0.92	0.97	0.94	0.90

With SVM as the ML algorithm, both ML_1_ and ML_2_ showed improved performances from the first to second rounds (R, P, F1) = (0.87, 0.97, 0.92) to (0.85, 1.00, 0.92) and (0.95, 0.67, 0.79) to (0.88, 0.98, 0.93), respectively. When R was set as 0.99, P improved from 0.70 to 0.82, and from 0.45 to 0.83, for ML_1_ and ML_2_, respectively.

With LR as the ML algorithm, ML_1_ and ML_2_ showed similar improved performances from the first to second rounds. In particular, when R was set as 0.99, P improved from 0.81 to 0.84, and from 0.60 to 0.90, for ML_1_ and ML_2_, respectively.

**FIGURE 7:**
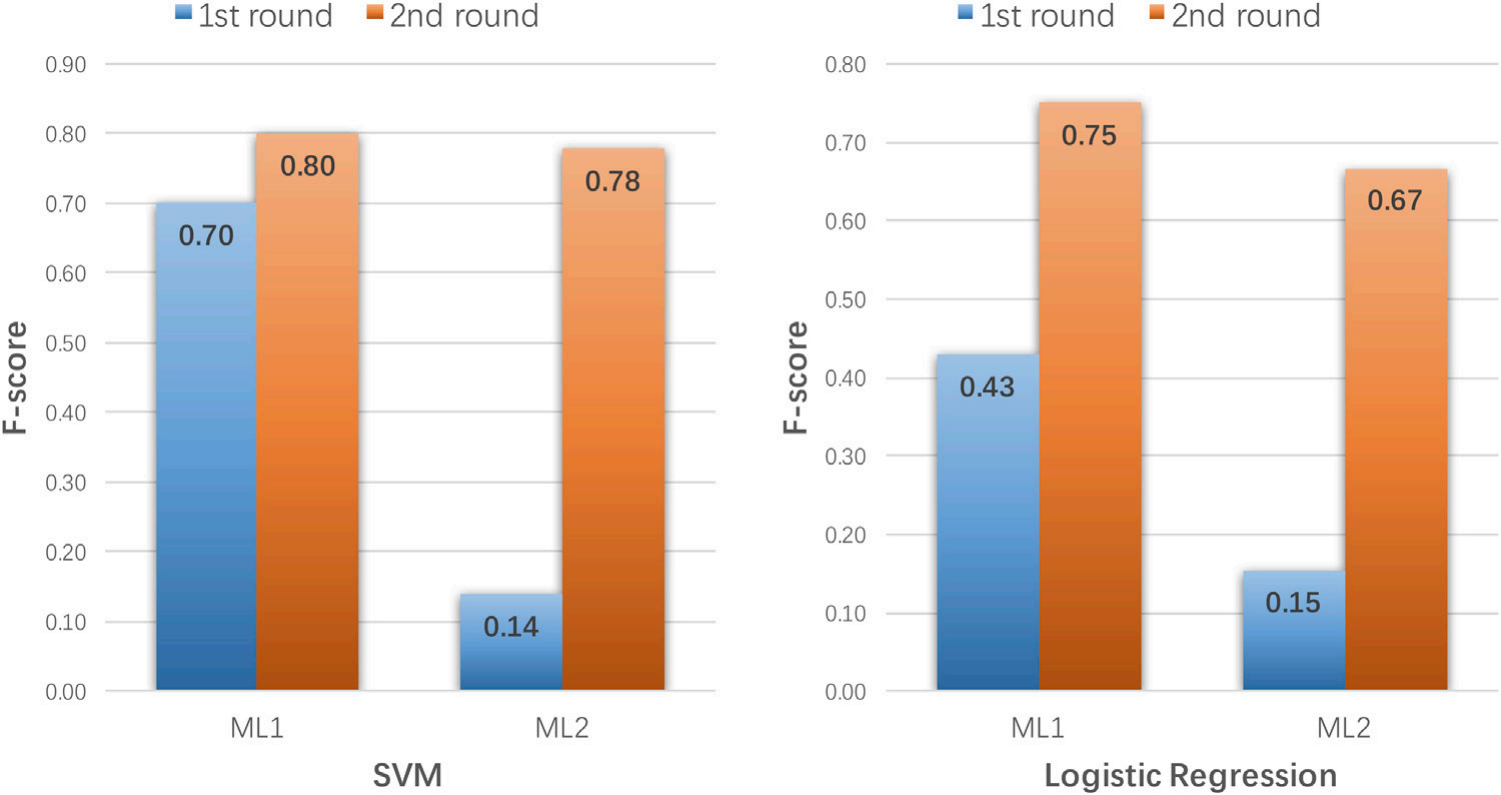
Performance of ML_1_ and ML_2_ on uncertainty samples with SVM and logistic regression as the machine learning (ML) methods.

To further illustrate why our AL method was better than the other AL methods at identifying uncertainty samples and to show how it helped to improve the traditional ML models, we analyzed the performances of ML_1_ and ML_2_ on the added uncertainty samples from the first and second rounds (Figure 7). When SVM was the ML method and the second round models were used to predict the misclassified samples, the F-score increased from 0.70 to 0.80 with ML_1_ and 0.14 to 0.78 with ML_2_. Similarly, using LR as the ML method, the F-score increased from 0.43 to 0.75 with ML_1_ and 0.15 to 0.67 with ML_2_. These results demonstrate that the second round models from our AL method showed significantly improved performances in predicting misclassified or vaguely classified samples from the first round.

### 3.3 Distribution Patterns of Uncertainty Samples, Random Negative Samples, and Training and Validation Samples

**FIGURE 8:**
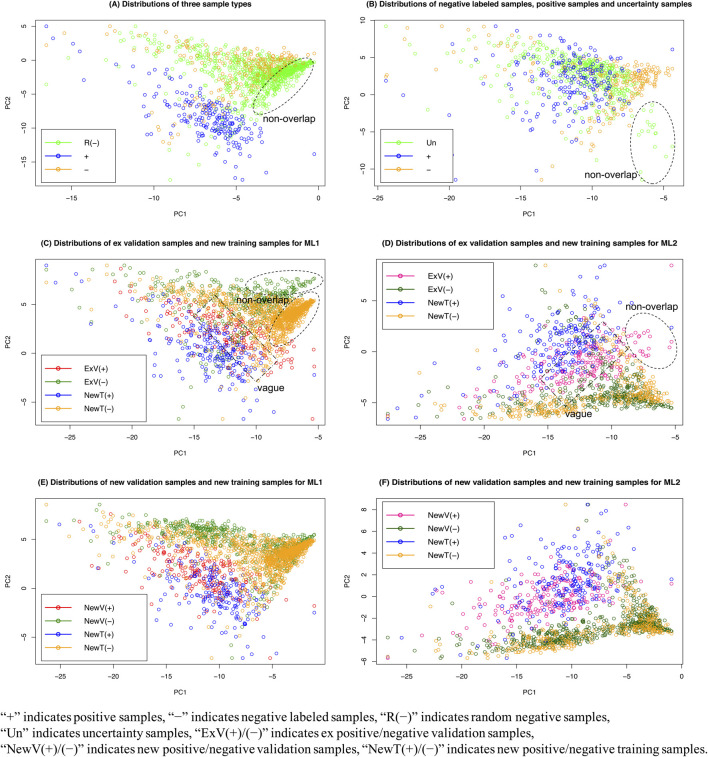
Distribution patterns of various sample types by principal component analysis.

We performed principal component analysis (PCA) of the various sample types, and the distribution patterns are shown in Figure 8. We expected the distribution patterns would provide insights into differences among the different sample sets and justify the sampling scheme of our AL method. Figure 8A shows the distributions of random negative samples negatively labeled and positively labeled abstracts. The positive samples are clearly different from the two types of negative sample sets, but a subset of random negative samples did not overlap with the negatively labeled abstracts. These data justify why two ML algorithms were needed for the two types of negative sample sets.

Figure 8B presents the distributions of negatively labeled and positively labeled abstracts and uncertainty samples. Some uncertainty samples were unlike both the positive and negative sample sets, which shows why both the training and validation data needed to be updated from the uncertainty samples.

Figure 8C–F displays the distributions of the training and validation sample sets before and after they were updated for the ML_1_ and ML_2_ algorithms. For ML_1_, which was built to differentiate random negative samples from positively labeled samples, the new positive/negative validation samples were very similar to the new positive/negative training samples (Figure 8E), but the ex-positive/negative validation samples were not (Figure 8C). For ML_2_, which was built to differentiating negatively labeled samples from positive samples, the training and validation samples had similar patterns (Figure 8D,F). Together, the results of these analyses further demonstrate the need to update both validation and training samples at the same time.

## 4 Discussion

In this study, we investigated how AL improved the performance of AL methods in DDI IR analysis. We recognized that the direct application of AL did not address several primary challenges in DDI IR from the literature. For instance, the vast majority of abstracts in PubMed are negative; the existing positive and negative labeled samples do not represent the general sample distributions, and potentially biased sample sets may arise during the uncertainty sampling process in an AL algorithm. Therefore, several novel AL sampling and ML schemes were developed to address these challenges in DDI IR analysis. In particular, random negative sampling was added as a part of AL since it has no expanse in the manual data label. Instead of one ML algorithm, we used two ML algorithms in an AL process and differentiated random negative samples from manually label negative samples. Further, both the training and validation samples were updated during the AL process to avoid or reduce biased sampling. Two supervised ML algorithms, SVM and LR, were used to investigate the consistency of our proposed AL algorithm. Given that the ultimate goal of DDI IR is to identify all DDI toxicity–relevant abstracts, a recall rate of 0.99 was set for developing the AL methods. Using our newly proposed AL method with SVM, the precision improved from 0.45 in the first round to 0.83 in the second round in differentiating the positive samples from the manually labeled negative samples, and from 0.70 to 0.82 in differentiating the positive samples from random negative samples. When LR was used in the AL, the precision improved from 0.60 to 0.90 in differentiating the positive samples from the manually labeled negative samples, and from 0.81 to 0.84 in differentiating the positive samples from random negative samples, in the first and second rounds, respectively. However, the other AL algorithms did not show improved precision largely because of biased samples that were caused by either the uncertainty sampling or differences between training and validation data sets. These sampling biases were further demonstrated in the PCA of distribution patterns.

To the best of our knowledge, our AL method is the first to integrate uncertainty sampling and random negative sampling. Our AL method allows two separate ML models to differentiate random negative samples and manually labeled negative samples, and the AL updates both training and validation samples. Together, these features contribute to its demonstrated success in DDI toxicity data IR.

However, cautions will be required when applying our AL methods to other DDI text mining tasks. In this study, text mining IR was focused on clinical drug safety DDI data, whereas other published DDI IR methods were focused on pharmacokinetic DDI data, so had different targeted samples. Moreover, clinical drug safety DDI–related articles have a smaller proportion in PubMed than DDI-relevant articles, even though DDI-relevant articles are only a very small fraction. This situation makes an unbalanced data composition for AL study. Our AL method addresses this problem and avoids the biased sampling by random negative sampling and validation set updating. Therefore, the performance of our AL method in DDI IR cannot be compared directly with the other DDI IR methods which focus on the existing DDI corpora. It has the capability to retrieve the information from the datasets with unbalanced samples.

## 5 Conclusion

Although our AL method integrated random negative sampling and uncertainty sampling, and performed well on clinical drug safety DDI IR systems, it can be further improved. In future work, we intend to adjust our method by training on different natural language processing methods and investigate its application in different DDI knowledge domains.

## Data Availability

The raw data supporting the conclusions of this article will be made available by the authors, without undue reservation.
